# Voice Disorders in Mucosal Leishmaniasis

**DOI:** 10.1371/journal.pone.0101831

**Published:** 2014-07-23

**Authors:** Ana Cristina Nunes Ruas, Márcia Mendonça Lucena, Ananda Dutra da Costa, Jéssica Rafael Vieira, Maria Helena de Araújo-Melo, Benivaldo Ramos Ferreira Terceiro, Tania Salgado de Sousa Torraca, Armando de Oliveira Schubach, Claudia Maria Valete-Rosalino

**Affiliations:** 1 Evandro Chagas National Institute of Infectious Diseases, Oswaldo Cruz Foundation, Rio de Janeiro-RJ, Brazil; 2 Department of Speech Pathology, Federal University of Rio de Janeiro, Rio de Janeiro-RJ, Brazil; 3 Federal University of Rio de Janeiro, Rio de Janeiro-RJ, Brazil; 4 Federal University of the State of Rio de Janeiro, Rio de Janeiro-RJ, Brazil; 5 Department of Otorhinolaryngology and Ophthalmology, Federal University of Rio de Janeiro, Rio de Janeiro-RJ, Brazil; The Ohio State University, United States of America

## Abstract

**Introduction:**

Leishmaniasis is considered as one of the six most important infectious diseases because of its high detection coefficient and ability to produce deformities. In most cases, mucosal leishmaniasis (ML) occurs as a consequence of cutaneous leishmaniasis. If left untreated, mucosal lesions can leave sequelae, interfering in the swallowing, breathing, voice and speech processes and requiring rehabilitation.

**Objective:**

To describe the anatomical characteristics and voice quality of ML patients.

**Materials and Methods:**

A descriptive transversal study was conducted in a cohort of ML patients treated at the Laboratory for Leishmaniasis Surveillance of the Evandro Chagas National Institute of Infectious Diseases - Fiocruz, between 2010 and 2013. The patients were submitted to otorhinolaryngologic clinical examination by endoscopy of the upper airways and digestive tract and to speech-language assessment through directed anamnesis, auditory perception, phonation times and vocal acoustic analysis. The variables of interest were epidemiologic (sex and age) and clinic (lesion location, associated symptoms and voice quality.

**Results:**

26 patients under ML treatment and monitored by speech therapists were studied. 21 (81%) were male and five (19%) female, with ages ranging from 15 to 78 years (54.5+15.0 years). The lesions were distributed in the following structures 88.5% nasal, 38.5% oral, 34.6% pharyngeal and 19.2% laryngeal, with some patients presenting lesions in more than one anatomic site. The main complaint was nasal obstruction (73.1%), followed by dysphonia (38.5%), odynophagia (30.8%) and dysphagia (26.9%). 23 patients (84.6%) presented voice quality perturbations. Dysphonia was significantly associated to lesions in the larynx, pharynx and oral cavity.

**Conclusion:**

We observed that vocal quality perturbations are frequent in patients with mucosal leishmaniasis, even without laryngeal lesions; they are probably associated to disorders of some resonance structures (larynx, pharynx and nasal and oral cavities) or even to compensation mechanisms caused by the presence of lesions in the upper airways and digestive tract.

## Introduction

Leishmaniasis is a public health problem in 98 countries and 3 territories on 5 continents. Tegumentary leishmaniasis (TL) is found in several countries with annual registration of 1 to 1.5 million cases [1].

American tegumentary leishmaniasis (ATL) is endemic in Brazil with 21.981 cases registered in 2010, of which 86 in Rio de Janeiro [2]. Mucosal leishmaniasis (ML) occurs from blood or lymph spread of a cutaneous lesion, even after several years from the primary skin lesion has healed [3], [4], [5]. The nasal mucous, isolated or associated to other locations is involved in almost all ML cases. The most likely places are the mucosa of the cartilaginous septum, lateral walls, nasal vestibule and head of the inferior turbinate. Other affected areas are the palate, lips and tongue [3], [6], [7]. The most common complaints of nasal impairment are obstruction, epistaxis, rhinorrhea and crusts. When the pharynx is affected the most common complaint is odynophagia and when the larynx is affected the complaints are dysphonia and cough [8]. It is believed that mucosal lesions increase when not treated, although there are some reports of possible spontaneous healing of those lesions [6], [9]. Even when treated, the lesions can leave sequelae such as retraction of the nasal pyramid, perforation of the nasal septum or palate and destruction of the uvula [10], these can interfere with the process of swallowing, breathing, voice and speech, requiring rehabilitation [11].

Voice production is directly dependent on the adequate functioning of the upper respiratory and digestive tracts. The layered structure of the vocal folds favors flexibility and the formation of mucosal waves, responsible for sound production in the glottis. In addition to this structure, the resonance boxes (larynx, pharynx, oral and nasal cavities and paranasal sinuses) are responsible for voice production and projection. The degree of voice perturbation depends on the extent of the disease, lesion location and compensation mechanisms developed [12]–[14].

The objective of the present study is to describe upper respiratory and digestive tracts anatomical characteristics and voice quality of a group of active ML patients.

## Materials and Methods

A transversal study was conducted in a cohort of 26 ML patients monitored at the Laboratory for Leishmaniasis Surveillance of the Evandro Chagas National Institute of Infectious Diseases - Fiocruz, Rio de Janeiro, Brazil, between 2010 and 2013. The parasitological diagnosis was established through one or more methods (direct examination by scraping or imprint, histopathology, culture, immunohistochemistry or Protein Chain Reaction) [15].

All patients were submitted to anamnesis and otorhinolaryngologic examination by 30° rigid nasal endoscopy and through 70 degrees Karl Storz rigid videolaryngoscope (Tuttlingen, Germany), to evaluate presence and localization of mucous lesions.

The speech language evaluation included: directed anamnesis for the symptoms (dysphonia, nasal obstruction, odynophagia and dysphagia); auditory perceptive evaluation through the GRBAS[14] scale that evaluates grade of hoarseness (G), considering level of roughness (R), breathiness (B), asthenia (A) and strain (S), which are classified from 0 to 3, with 0 = normal, 1 = slightl, 2 = moderate, and 3 = severe [16]; phonation time assessment with maximum phonation time (MPT) of the sustained vowel/a/ and ratio between voiced and voiceless fricatives (S/Z). Along of the voice acoustic analysis, the voice of the patients was recorded in quiet environment, directly in the computer for better capture of the voice by Vox Metria software (CTS Informática, Pato Branco, Brazil). A Plantronix-model A-20 microphone was used, with a 10 cm mouth-microphone distance, during the emission of the/e/vowel at normal condition [17]. The parameters Jitter that indicates the variability of the fundamental frequency perturbation in the short term, with normal pattern up to 0.6%; Shimmer, that indicates the variability of the amplitude of the vocal note in the short term and with normal values up to 6.5% and measures of Glottal to Noise Excitation Ratio (GNE), which is an acoustic measure to assess noise in a pulse train that is typically generated by the oscillation of the vocal folds, with normal values above 0.5 (dimensionless) were analyzed in the present study. Voice quality was diagnosed by perceptive and acoustic evaluation.

The frequencies of the categorical variables were calculated. The continuous variable age was examined through mean ± standard deviation. The difference between age means was evaluated according to voice quality and dysphonia by the t test. The association between categorical variables was investigated by Fisher exact test. A significance level of 5% was considered. The Statistical Package for Social Sciences version 16.0 was used for the data analysis.

All the participants signed an informed free consent form. In the case of the minors/children enrolled in our study, we obtained written informed consent from the guardians on behalf of them. This project was approved by the Ethics in Research Committee CEP-INI under protocol number 0043.0.009.000-10.

## Results

26 patients under ML treatment were studied. 81% (n = 21) were male, with age between 15 and 78 years (mean = 54.5±15.0 years), of these patients, four were smokers and seven alcohol user.

Fourteen (53.6%) patients had lesion in a single upper respiratory and digestive tracts mucous site, five (19.2%) in two sites, five (19.2%) in three sites and two (7.7%) in four sites with the following distribution: 88.3% (n = 23) in the nasal cavity, 38.4% (n = 10) oral cavity, 34.5% (n = 9) pharynx and 19.2% (n = 5) larynx (Table 1). Twenty three patients reported complaints: nasal obstruction (73.1%), dysphonia (38.5%), odynophagia (30.8%) and dysphagia (26.9%).

**Table 1:** Location of the mucous lesions in 26 patients with mucous leishmaniasis.

Anatomical structure affected- alone or in combination	Number of patients
Nose	12
Oral cavity	1
Pharynx	1
Nose + Pharynx	2
Nose + Oral cavity	2
Larynx + Pharynx	1
Nose + Larynx + Oral cavity	2
Nose + Oral cavity + Pharynx	3
Nose + Oral cavity + Larynx + Pharynx	2

The speech-language evaluation showed that 88.5% had voice quality perturbation. The parameters analyzed MPT, S/Z, Jitter, Shimmer and GNE are represented in diagram 1. The results of the auditory perceptive evaluation using the GRBAS scale are shown in Table 2.

**Table 2:** Degree of voice perturbation in the GRBAS* scale in 26 mucosal leishmaniasis patients, Evandro Chagas Clinical Research Institute, Oswaldo Cruz Foundation.

Degree/Scale	G		R		B		A		S	
	n	%	n	%	n	%	n	%	n	%
**Normal**	3	11.5	7	26.9	13	50	0	0	11	42.3
**Light**	10	38.5	12	46.2	9	34.6	0	0	10	38.5
**Moderate**	10	38.5	4	15.4	4	15.4	0	0	5	19.2
**Severe**	3	11.5	3	11.5	0	0	0	0	0	0

*GRBAS scale. G - hoarseness, R - roughness B - breathiness; A - asthenia; S - strain.

Patients with dysphonia complaints presented a greater proportion of lesions of the pharynx (80%, p<0.001), oral cavity (70%, p = 0.015) and larynx (50%, p = 0.004), when compared to the patients without complaints. The five patients with lesions of the larynx reported dysphonia and voice disorders complaints.

No significant associations were found between voice quality perturbations and age, sex, smoking, alcohol consumption, and place of the lesions.

## Discussion

In this study we evaluated the voice quality of 26 patients with ML diagnosis, predominantly males and with higher age, as expected [1], [7].

In accordance with other authors [1], [8], [18], the nasal cavity was the most affected structure and, consequently the major complaint was nasal obstruction. Although dysphonia was the second most frequent symptom, paradoxically the larynx was the less affected structure. Additionally, dysphonia was also associated to lesions in the pharynx and oral cavity, besides the larynx. Dysphonia in mouth breathers without infectious diseases has already been reported [19]. The nasal obstruction favors mouth breathing which promotes a series of postural, muscle tone and cervical tension changes. This facilitates the entry of air but interferes with phonation and cause a resonance imbalance [19], [20].

The observation of voice quality perturbations during the auditory perception and acoustic evaluation with higher frequency than the patients’ dysphonia complaint suggests that these evaluations are capable of identifying asymptomatic alterations.

All the patients with larynx lesions presented dysphonia and voice quality perturbations although these disorders were also present in patients with lesions of the pharynx, nasal and oral cavities. However, dysphonia in ML patients had not yet been associated to lesions of other anatomical sites than the larynx [21].

In a previous study, we described that clinical healing of the larynx tuberculosis lesion was not enough for a complete recovery of the patients’ voice quality [17]. It would be interesting to verify if those voice disorders found in ML patients are present even after a favorable response to therapy.
